# Non-Ionic Deep Eutectic Liquids: Acetamide–Urea Derived Room Temperature Solvents

**DOI:** 10.3390/ijms20122857

**Published:** 2019-06-12

**Authors:** Subramanian Suriyanarayanan, Gustaf D. Olsson, Subban Kathiravan, Natacha Ndizeye, Ian A. Nicholls

**Affiliations:** Bioorganic & Biophysical Chemistry Laboratory, Linnaeus Centre for Biomaterials Chemistry, Department of Chemistry & Biomedical Sciences, Linnaeus University, SE-391 82 Kalmar, Sweden; gustaf.olsson@lnu.se (G.D.O.); suppan.kathiravan@lnu.se (S.K.); natacha.ndizeye@lnu.se (N.N.)

**Keywords:** deep-eutectic solvent, flickering cluster, acetamide–urea

## Abstract

A family of non-ionic deep eutectic liquids has been developed based upon mixtures of solid *N*-alkyl derivatives of urea and acetamide that in some cases have melting points below room temperature. The eutectic behaviour and physical characteristics of a series of eleven eutectic mixtures are presented, along with a molecular dynamics study-supported hypothesis for the origin of the non-ideal mixing of these substances. Their use as solvents in applications ranging from natural product extraction to organic and polymer synthesis are demonstrated.

## 1. Introduction

Liquids, and in particular organic liquids, are used extensively as solvents and dispersants in a wide variety of processes, e.g., synthetic chemistry, material synthesis or fabrication, the formulation of foods, cosmetics and pharmaceuticals, separation or partitioning, heat transfer, and as detergents or cleaners. The physicochemical properties of a liquid, or a liquid mixture, govern the solvent or dispersant properties of the mixture, which in turn defines their operational range in a given application. Physicochemical properties such as polarity, dielectricity, hydrogen bonding, heat capacities and ionization capacities, etc. are inherent to a given liquid or liquid mixture, as are its toxicities. 

The significance of liquid solvents and dispersants for processes important to society has driven the search for new liquids with solvation or dispersant properties better suited to particular applications, some examples of such liquids include supercritical carbon dioxide and ionic liquids [1]. Ionic liquids are examples of eutectic liquids [2], these are comprised of two or more substances with a particularly low melting point relative to those of the constituent components. Those fulfilling this requirement and with a melting point lower than 100 °C are often referred to as a deep eutectic liquid or solvent (DES) [3,4,5,6,7,8,9,10,11,12,13,14,15,16]. Over the past few decades the properties of ionic liquids have been extensively explored in areas as diverse as chemical synthesis [17] and drug delivery [18]. The unique molecular-level environments for reactions provided by ionic liquids have afforded excellent results in a range of synthesis applications [19]. However, their high cost, high levels of toxicity and poor biodegradability and (for some applications) high conductivities are factors that make their use undesirable, or impossible. Alternatives to ionic liquids that are devoid of these problems would undoubtedly be of value.

Soviet scientists searching for alternatives to imported fertilizers identified the first non-ionic deep eutectic liquid [20], which was based upon a mixture of the biologically and environmentally benign substances urea and acetamide [mp 406 K and 353 K respectively, eutectic (33 wt% urea/67 wt% acetamide) = 329 K]. Identifying other non-ionic DESs is naturally of interest, as is the exploration of the fundamental properties of non-ionic DESs and their use in mediating molecular level (Ångström- and nanometer-scale) events in synthesis and the development of new materials. Moreover, the low toxicities of acetamide, which has been used as a fertilizer and even found in interstellar space, [21] and urea together with their availability from renewable sources provides further motivation for investigation of the use of this or related non-ionic DESs as alternatives to traditional ionic liquids and other environmentally problematic organic solvents.

## 2. Results and Discussion

**Figure 1:**
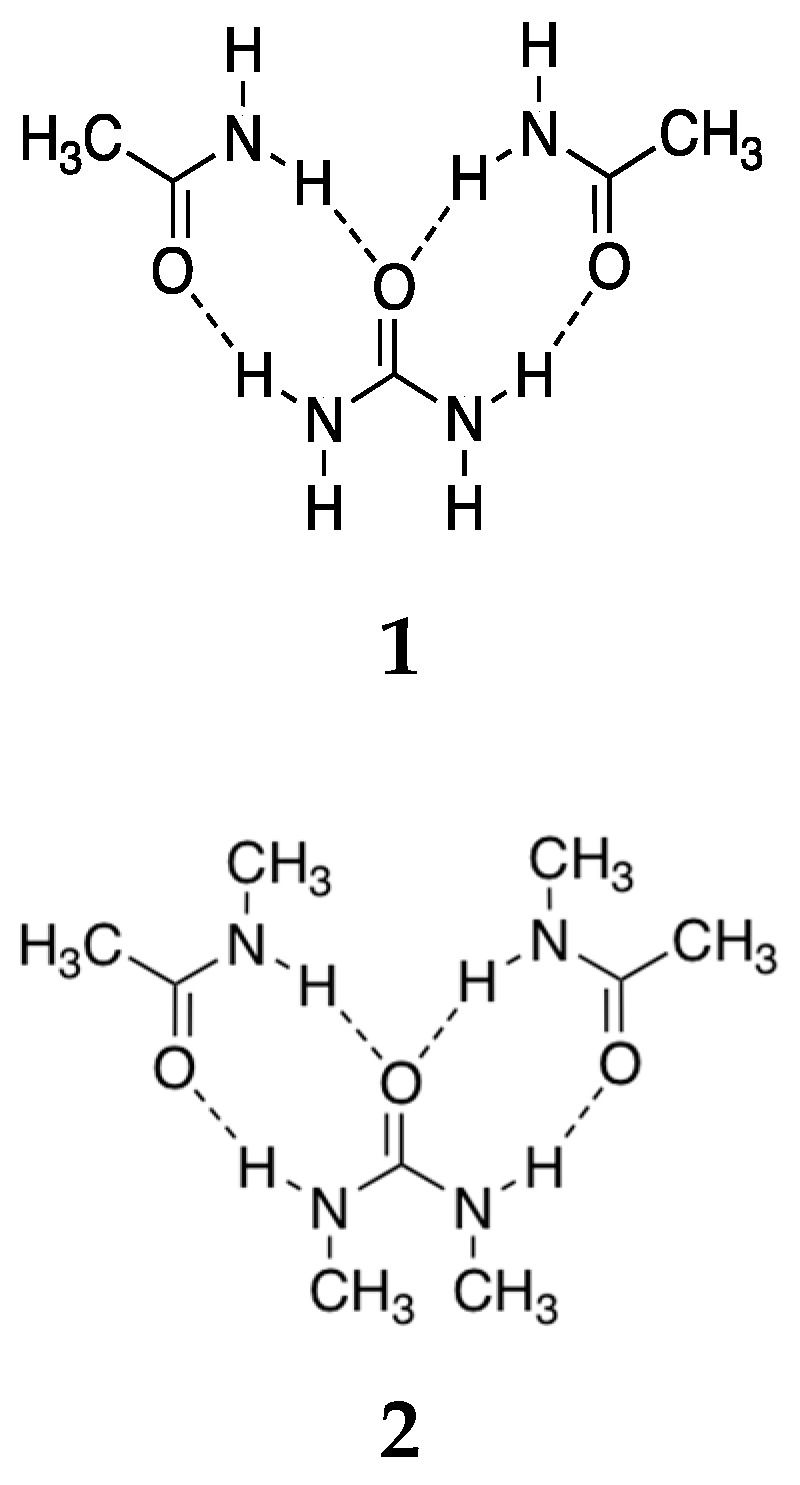
Structure of suggested flickering cluster-like complexes of (**1**) A–U and (**2**) NMA–NN’DMU.

To explore the mechanisms underlying the DES-behavior of this system we undertook a series of all-atom molecular dynamics simulations using a total ensemble of 1000 molecules of acetamide (Scheme S1, A, 650) and urea (Scheme S1, U, 350) at 343 K, equilibration for 22 ns (nPT) and a production run of 2 ns (nVT). Analysis of hydrogen bonding interactions in the mixture (Table 1, Table S1) provided evidence of a preferential hydrogen bonding interaction between urea and acetamide at the expense of urea–urea and acetamide–acetamide interactions as reflected in the hydrogen bond occupancies throughout the simulation. This observation suggested the presence of flickering cluster-like species formed through the interaction of acetamide and urea, which can provide a possible basis for the observed deviation from Raoult’s Law, whereby the interactions between the acetamide and urea are stronger than between the clusters (Figure 1 (**1**)) This is in contrast to a study by Das et al., [22] who argued for a spatially and dynamically homogenous system based upon an observed Gaussian distribution when using a total of 512 molecules and a production run of 20 ns. Interestingly, the stoichiometry of the eutectic point alludes to a two (acetamide) to one (urea) complex. While stable complexes of this nature were not observed in our molecular dynamics study, transient binary and ternary complexes were seen. 

We perceived that the bias in favor of acetamide–urea interactions could be exploited to test the validity of the proposed basis for deviation from Raoult’s law and, potentially, to produce alternative non-ionic DESs. To enhance this bias at the expense of interaction between complexes, we envisaged using *N*-methyl substituents to remove extra H-bond donors. Accordingly, a series of acetamide and urea-based derivatives was then explored in attempts to develop other DESs, Table 2.

Phase diagrams were produced for the acetamide–urea and nine novel binary mixtures (Figure S1) and from these, eutectic points were identified along with their corresponding mixture stoichiometries. Upon comparison with the acetamide–urea system (A–U), the incorporation of a single methyl substituent on the urea (A–NMU) was seen to lower the eutectic point and lead to a change in stoichiometry from 2:1 to 1:1. Interestingly, blocking one hydrogen bond donor site on each of urea’s nitrogen atoms, A–NN’DMU, produced a similar result both in terms of temperature and stoichiometry, while blocking both on the same nitrogen, A–NNDMU, results in a higher eutectic point and a quite different stoichiometry, 4:1

We then examined the influence of a methyl substituent on the acetamide (NMA), where in conjunction with *N*-methylurea (NMU) they produced a sub-room temperature (287 K) non-ionic DES with a 4:1 stoichiometry. The presence of an additional methyl (NN’DMU), removing a second hydrogen bond donor also produced a sub-room temperature (285 K) non-ionic DES though with a 2:1 stoichiometry. In this case, the stoichiometry reflects an idealized 2:1 complex, where compared to the A–U case (Figure 1), the possibilities for hydrogen bond interactions between complexes is removed through the presence of the hydrophobic methyl substituents blocking all outward projected hydrogen bond donors, Scheme S1. Our rationale for the underlying mechanisms steering the eutectic behavior of these systems found support in the report of a caprolactam–urea system (1:1) displaying deep eutectic behavior (18 °C) that appeared during the course of these studies [23]. Interestingly, no eutectic mixture was obtained in studies on NMA–NNDMU (data not shown) where only one of the urea nitrogens can donate a hydrogen bond. Systems where both components were urea derivatives also produced DESs, though none with eutectic points near room temperature, reflecting the replacement of the hydrophobic methyl of acetamide with the polar amide nitrogen.

An examination of the conductivities of the non-ionic DESs revealed them to be quite polar in character with values spanning the range ≈10–120 µScm^−1^ (Table 2), and with values decreasing with increasing numbers of H-bond donor-blocking methyl substituents. Our observation that these non-ionic DESs were somewhat viscose prompted a study of their viscosities (Table 2), which were observed to be in a range comparable to those of n-octanol and ethylene glycol, 6.2 [24] and 19.1 [25] cP at 22.4 °C, respectively. The solvation properties of the NMA–NMU system were explored (other systems are to be reported in detail elsewhere), and detailed in Table 3. A range of substances, inorganic and organic were readily solubilized. Of note is the maize-derived protein zein which has very limited solubility in water and organic solvents (except methanol) which had an appreciable solubility in the NMA–NMU DES.

**Scheme 1:**
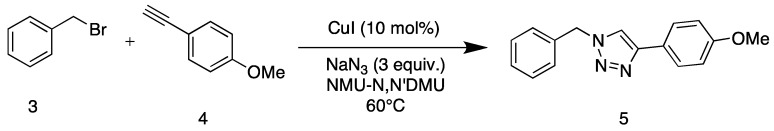
Copper catalyzed click reaction of benzyl bromide (**3**) and 4-methoxy phenyl acetylene (**4**) to yield 1-benzyl-4-(4-metoxyphenyl)-1*H*-1,2,3-triazole (**5**).

**Figure 2:**
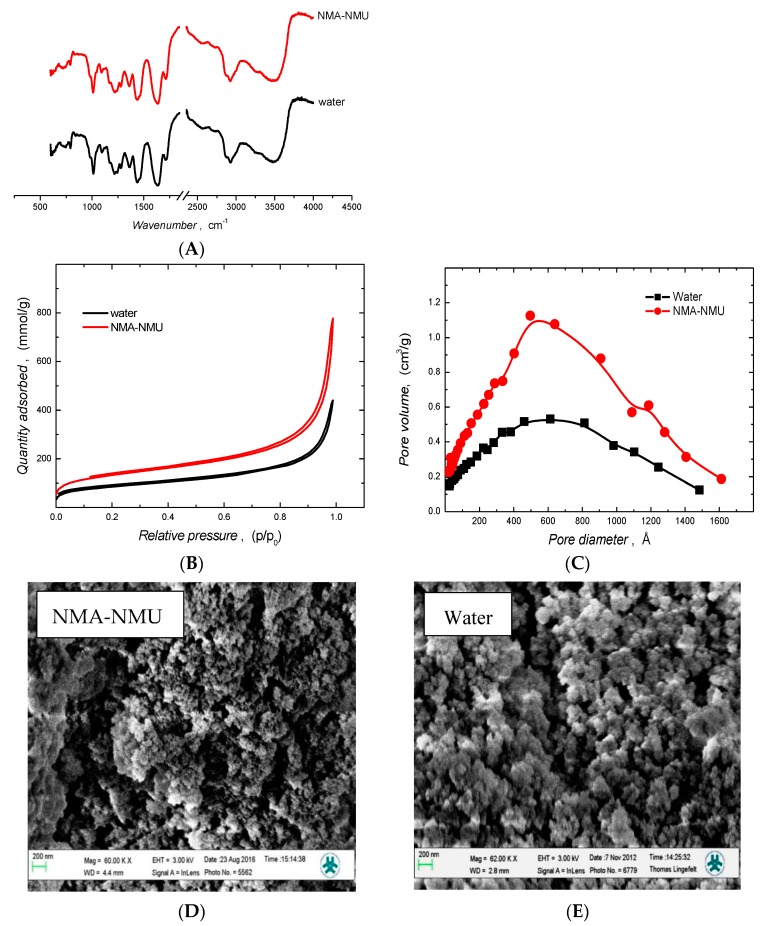
(**A**) Infrared spectra. (**B**) Nitrogen adsorption-desorption isotherms and (**C**) pore distribution plot (desorption) observed from BET adsorption isotherm experiments. (**D**,**E**) Scanning electron micrographs, respectively, of MAA–BAP copolymer synthesized in NMA–NMU eutectic system and water.

We then explored the stabilities of these liquids by repeated heating and cooling over the range from 10 °C below their respective eutectic points to 150 °C. In each case no change in deviation in eutectic point was observed after ten cycles, which suggests the potential of these DESs for use as heat exchangers over suitable temperature intervals. Initial efforts to explore the potential for using these solvents in a range of applications included use as an alternative to chloroform for the extraction of betulin from birch bark [32]. In the case of the sub-room temperature NMA–NMU, 26% wt/wt was obtained, as compared to 30% wt/wt using chloroform (Figure S2). The utility of these novel solvents in organic synthesis was demonstrated, first by the use of NMA–NMU as a porogen for the synthesis of copolymers of methacrylic acid and bisacryloylpiperazine (Figure 2), and, secondly, as a solvent for the click reaction (Scheme 1 and Figure S3). The anticipated products were obtained in yields comparable to those using conventional solvents (see Section 3.2.6 and Section 3.2.7).

## 3. Materials and Methods 

### 3.1. Chemicals

Acetamide (A), urea (U), *N*-methylacetamide (NMA), *N*-methylurea (NMU), *N*,*N*′-dimethylurea (NN’DMU) and *N*,*N*-dimethylurea (NNDMU) (see Scheme S1 in ESI), methacrylic acid (MAA), 1,4-bis(acryloyl)piperazine (BAP), 2,2′-azobis(2-methylpropionamidine) dihydrochloride (ABAH), potassium chloride, silica gel were all purchased from Sigma-Aldrich (Steinheim, Germany) and stored in vacuum desiccator. MAA was vacuum distilled and stored at 4 °C. ABAH was recrystallized in acetone–water mixture (1:1, *v*/*v*). Methanol, acetone and chloroform were of analytical grade obtained from Chemtronica (Stockholm, Sweden) and dried over 4 Å molecular sieves. De-ionized water (resistance value < 18.2 MΩ) collected from a Milli-Q gradient water filtration system (Millipore, MA, USA), was used in this study. 

### 3.2. Instrumentation and Protocols 

#### 3.2.1. Molecular Dynamics Study of Acetamide (A)-urea (U) Interaction

System setups, parameterizations and simulations were performed using the AMBER tools 1.5 and Amber 14 software suites [33]. Partial atomic charges were assigned to structures using the AM1-BCC charge method. Random initial starting configurations were produced using PACKMOL, [34,35] parameterized using the amber and the general amber force fields [36,37]. Langevin dynamics with a collision frequency of 1.0 picoseconds^−1^ and isotropic positional scaling with a pressure time relaxation of 2.0 picoseconds were used to keep temperature and pressure constant. Periodic boundary conditions were employed in combination with a 9 Å non-bonded interaction cut-off. Long-range electrostatics were treated using the particle mesh Ewald summation method, long-range van der Waals interactions were treated using a continuum model correction to energy and pressure. All bonds to hydrogen atoms were constrained using the SHAKE algorithm. Final production phase trajectories were analyzed using the PTRAJ module hbond included in AMBER tools 1.5 with 3.0 Å distance and 60° angle cut-off values respectively. 

The study consists of a simulation of a 0.65:0.35 liquid mixture system of acetamide (A): urea (U) at 70 °C (343 K) well above the eutectic point temperature. Hydrogen bond analyses were performed considering all hydrogens as potential donors and heteroatoms (N and O) as acceptors. All hydrogen bonding events were summarized and averaged with respect to both acetamide (Table S1), and to confirm the relationship, with respect to urea (Table S2). Average hydrogen bond occupancies per molecule of A and U were calculated based upon Table 1.

#### 3.2.2. General Procedure for Preparation of Eutectic Mixtures

Calculated amounts of the components A and B, where the melting point of component A is less than component B, were added in a 50 mL shallow bottomed flask fitted with ground-joint thermometer. The mixture was heated in an oil bath (for temperatures > 80 °C) or water bath, at a temperature 5 °C above the melting point of component A. After the melting of component A, and after dissolution of component B, the mixture was then allowed to equilibrate for 10 min with stirring until a clear homogenous phase was obtained. The mixture was then slowly cooled to 10 °C below the observed freezing temperature. After 10 min, mixture was again heated to determine the melting temperature of the mixture. This process was repeated three times and the average value was taken. For accurate values, an aliquot of the mixture (5 μL) in liquid state was loaded in the capillary tubes (using a long needle syringe) and placed in a melting point apparatus to determine the melting and freezing temperature of the mixture. 

The ratio of the components in the mixture was varied to construct the phase diagrams for the binary mixtures of A and B using the freezing points and melting point curve. The lowest melting temperature in the phase diagram denotes the eutectic point of the binary mixture. 

#### 3.2.3. Characterization of Eutectic Mixtures

The conductivities of the euctectic mixtures were measured using a CDM 210 conductivity meter from MeterLab^TM^, Radioameter Analytical (Loveland, CO, USA) at temperature slightly above (5 °C) their eutectic point. The probe was calibrated with different concentrations of KCl(*aq*) at 20 °C and stored in deionized water prior to the measurements. 

The viscosities of the eutectic systems were determined with a Brookfield viscometer, DV-II+Pro (Middleboro, MA USA). Measurements were carried out at 100 rpm/5 °C above the eutectic temperatures. 

#### 3.2.4. Synthesis of Methacrylic Acid (MAA)–bisacryloylpiperazine (BAP) Co-Polymer

A copolymer of MAA and BAP was synthesized in NMA-NMU. To a solution of MAA (12 mmol) and BAP (55 mmol) in NMA-NMU (1.5 mL). The pre-polymerization mixture was saturated with nitrogen for 5 min to remove dissolved oxygen. The solution was then brought to the polymerization temperature (70 °C) before addition of 2,2′-azobis(2-amidinopropane) dihydrochloride (ABAH) initiator. The polymerization reaction mixture was maintained at 70 °C for 6 h using a heated oil bath. Following polymerization, the resultant bulk polymer was ground with a mortar and pestle before sieving to yield polymer particles of 63–125 μm size fraction. The polymer particles (~1 g) were slurry packed into an HPLC column and washed from unreacted monomers and porogen following a procedure reported by Karlsson et al. [38]. The polymer particles were emptied from the column and air-dried at ambient temperature prior to physical studies.

#### 3.2.5. Characterization of Polymers 

##### Brunauer-Emmett-Teller (BET) and Barrett-Joyner-Halenda (BJH) Analyses 

To determine pore volumes and surface areas of polymer particles, BET and BJH analyses were performed using an ASAP 2400 instrument (Micromeritics, Norcross, GA, USA). Samples were degassed for at 50 °C 24 h to remove adsorbed gases and moisture. BET surface areas were calculated from the adsorption data using 0.162 nm^2^ as the molecular cross-sectional area for adsorbed nitrogen molecules. The BJH method was applied to calculate the pore size distributions from desorption branches of the isotherms. 

##### Scanning Electron Microscopy (SEM) Analysis 

Prior to SEM analysis, polymer particles were deposited on black carbon tape attached to alumina stubs and were then coated with a thin layer of platinum by a platinum sputtering unit (LEICA EM SCD 500). These polymer particles were inserted in the Field Emission SEM (Leo 1550 Gemini) and scanned with an electron beam at 3 kV.

##### Fourier Transform infrared Spectrometry (FT-IR) Analysis

FT-IR analyses were performed using an Agilent Cary 630 FT-IR Spectrometer equipped with KBr optics and complementary Diamond attenuated total reflectance (ATR) sampling accessory. The Diamond ATR accessory uses a type IIa diamond crystal, where a small amount of ground polymer powders is placed to be measured. This system is configured with the Agilent MicroLab FT-IR Software for collecting a background spectrum and a sample spectrum. The samples were recorded within 400–4000 cm^−1^ range with 32 scans and 4 cm^−1^ resolutions.

#### 3.2.6. Extraction of Betulin from Birch Bark

Betulin was extracted based upon a procedure reported elsewhere [32]. Briefly, 500 mg of the bark was soaked in 15 mL of either NMA-NMU eutectic solvent at 60 °C or chloroform at reflux for 30 min. In the case of the chloroform extraction, the bark was then removed by filtration, washed with excess of chloroform solvent and the extract was passed through a short silica gel column (10 cm) to remove tan impurities. The collected effluent was evaporated to dryness in vacuo to yield betulin (150 mg, 30%, wt/wt). For the extraction using NMA-NMU, water (45 mL) was added and the aqueous solution extracted with ethyl acetate (60 mL). The organic phase was passed quickly through short silica gel column, dried over Na_2_SO_4_ and evaporated in vacuum to yield the betulin (129 mg, 26%). In both cases, product purity was confirmed by ^1^H-NMR, FT-IR. Melting point of the extracted betulin was 255 °C.

#### 3.2.7. General Procedure for Copper Catalyzed Click Reaction in Eutectic Mixture

To a capped vial equipped with a stirring bar containing NMU–NN’DMU (1 g, NMU/*N*,*N*′-DMU 1:1% wt/wt) were added benzyl bromide **3** (1 equiv.), 4-methoxy phenyl acetylene **4** (1 equiv.), CuI (0.1 equiv.) and sodium azide (3 equiv.) and the reaction was stirred for 3 h at 60 °C (see Scheme 1). Water (10 mL) was added to terminate reaction and the mixture was extracted with EtOAc (3 × 5 mL). The organic phase was dried over Na_2_SO_4_, filtered, evaporated and the crude product was subjected to column chromatography (heptane-ethyl acetate, 6:4) to yield the 1,4-substituted 1,2,3-triazole **5** as a colorless solid (99%). 

*1-benzyl-4-(4-metoxyphenyl)-1H-1,2,3-triazole* (**5**). Using the general procedure, compound **5** was isolated in 99% yield (colorless solid); ^1^H NMR (500 MHz, CDCl_3_): δ 7.63 (d, *J* = 5 Hz, 2H), 7.50 (s, 1H), 7.28 (d, *J* = 10 Hz, 2H), 7.20 (d, *J* = 5 Hz, 2H), 6.84 (d, *J* = 10 Hz, 2H), 5.44 (s, 2H), 3.72 (s, 3H). ^13^C NMR (125 MHz, CDCl_3_): δ 159.6, 148.1, 134.8, 129.1, 128.7, 128.0, 127.0, 123.3, 118.8, 114.2, 55.3, 54.2. These spectroscopic data correspond to previously reported data [39].

## 4. Conclusions

In summary, we describe here the first examples of sub-room temperature non-ionic deep eutectic liquids and demonstrate their use as alternatives to traditional organic solvents in natural product extraction and in organic and polymer synthesis. The low cost and often environmentally benign nature of these novel liquids, relative to ionic liquids and many traditional organic solvents, together with their unique properties highlight their potential for use in a wide range of applications [14].

## 5. Patents

Swedish patent application SE-1850195-7 (2018).

## Figures and Tables

**Table 1:** Sum of all averaged hydrogen bond occupancies.

	A ^§^	U ^§^
A	40.3	65.8
U	–	50.9

^§^ Percentage of time that hydrogen bond donor and acceptor sites are occupied throughout the simulation (see Table S1).

**Table 2:** Non-ionic deep eutectic mixtures comprised of components A and B where the general structures of A is either R^1^R^2^N-CO-CH_3_ or R^3^R^4^N-CO-NR^5^R^6^ and that of B R^7^R^8^N-CO-NR^9^R^10.^

System ^a^	R ^1^	R ^2^	R ^3^	R ^4^	R ^5^	R ^6^	R ^7^	R ^8^	R ^9^	R ^10^	Eutectic Point °C	Composition A:B ± 3	Conductivity μS/cm ^b^	Viscosity cP ^c^
A–U	H	H	-	-	-	-	H	H	H	H	56 ± 2	65:35	126	10.8
A–NMU	H	H	-	-	-	-	CH_3_	H	H	H	42 ± 3	50:50	30	9.2
A–NNDMU	H	H	-	-	-	-	CH_3_	CH_3_	H	H	68 ± 3	80:20	n.d.	8.5
A–NN’DMU	H	H	-	-	-	-	CH_3_	H	CH_3_	H	43 ± 3	50:50	64	10.1
NMA–NMU	CH_3_	H	-	-	-	-	H	H	CH_3_	H	14 ± 2	80:20	12	7.1
NMA–NN’DMU	CH_3_	H	-	-	-	-	CH_3_	H	CH_3_	H	12 ± 4	70:30	25	7.2
U–NMU	-	-	H	H	H	H	CH_3_	H	H	H	61 ± 2	70:30	27	14.8
U–NN’DMU	-	-	H	H	H	H	CH_3_	H	CH_3_	H	69 ± 3	70:30	n.d.	12.7
NMU–NNDMU	-	-	CH_3_	H	H	H	CH_3_	CH_3_	H	H	76 ± 4	80:20	n.d.	8.1
NMU–NN’DMU	-	-	CH_3_	H	H	H	CH_3_	H	CH_3_	H	49 ± 3	50:50	47	15.1

^a^ A = acetamide, U = urea, NMU = *N*-methylurea, NN’DMU = *N*,*N*′-dimethylurea, NNDMU = *N*,*N*-dimethylurea, NMA = *N*-methylacetamide. ^b^ measurements were performed at 5 °C above the eutectic temperatures, n.d. = not determined due to high temperature. ^c^ measured at 100 rpm/5 °C above the eutectic temperatures.

**Table 3:** Solubility of range of substance in NMA–NMU ni-DES compared to other solvents.

Solutes	Solvents (mg/mL)			
	Water (pH 5.9)	NMA–NMU	Methanol	Chloroform
NaCl [26]	360	12	15	Insoluble
Sucrose [27]	2000	9	10	Insoluble
Zein [28]	Insoluble	80	90	Insoluble
Biotin [29]	0.5	2	1	Insoluble
Theophylline [30]	8.3	5	12.5	9.1
*o*-phenanthroquinone [31]	0.4	50	2	2.5

## Supplementary Materials

Supplementary materials can be found at https://www.mdpi.com/1422-0067/20/12/2857/s1.
