# Supplementary material for: Non-Ionic Deep Eutectic Liquids: Acetamide–Urea Derived Room Temperature Solvents
Source: Int J Mol Sci. 2019 Jun 12;20(12):2857. doi: 10.3390/ijms20122857 (PMC6627579; doi:10.3390/ijms20122857)
Supplement: Supplementary file 1 [file ijms-20-02857-s001.pdf]

Supporting information

Non-Ionic Deep Eutectic Liquids: Acetamide–Urea Derived Room Temperature Solvents

Subramanian Suriyanarayanan *, Gustaf D. Olsson, Subban Kathiravan, Natacha Ndizeye and Ian A. Nicholls *

Bioorganic & Biophysical Chemistry Laboratory, Linnaeus Centre for Biomaterials Chemistry, Department of Chemistry & Biomedical Sciences, Linnaeus University, SE-391 82 Kalmar, Sweden;

* Correspondence: esusu@lnu.se (S.S.); ian.nicholls@lnu.se (I.A.N.)

**Scheme SI-1**	**2**
**Figure SI-1**	**3-4**
**Figure SI-2**	**5**
**Figure SI-3**	**6**
**Table SI-1**	**7**
**Table SI-2**	**8**

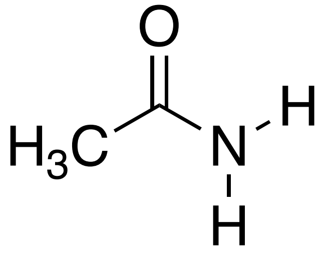

(1)

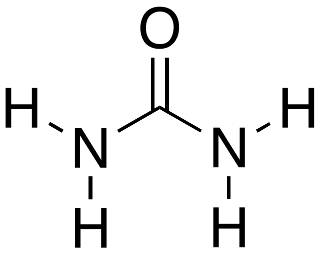

(2)

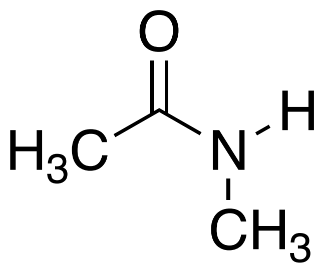

(3)

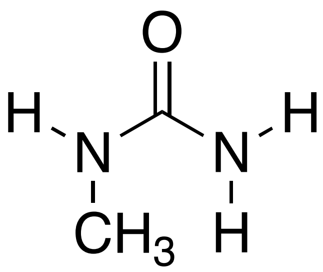

(4)

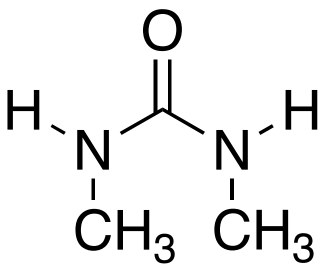

(5)

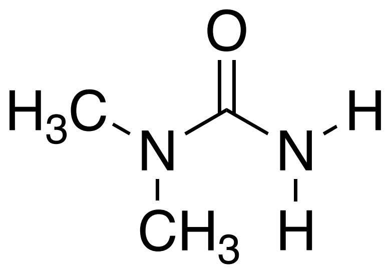

(6)

Scheme S1. Structures of amide derivatives used: (**1**) acetamide (A), (**2**) urea (U), (**3**) *N*-methylacetamide (NMA), (**4**) *N*-methylurea (NMU), (**5**) *N*,*N′*-dimethylurea (NN′DMU) and (**6**) *N*,*N*-dimethylurea (NN-DMU).

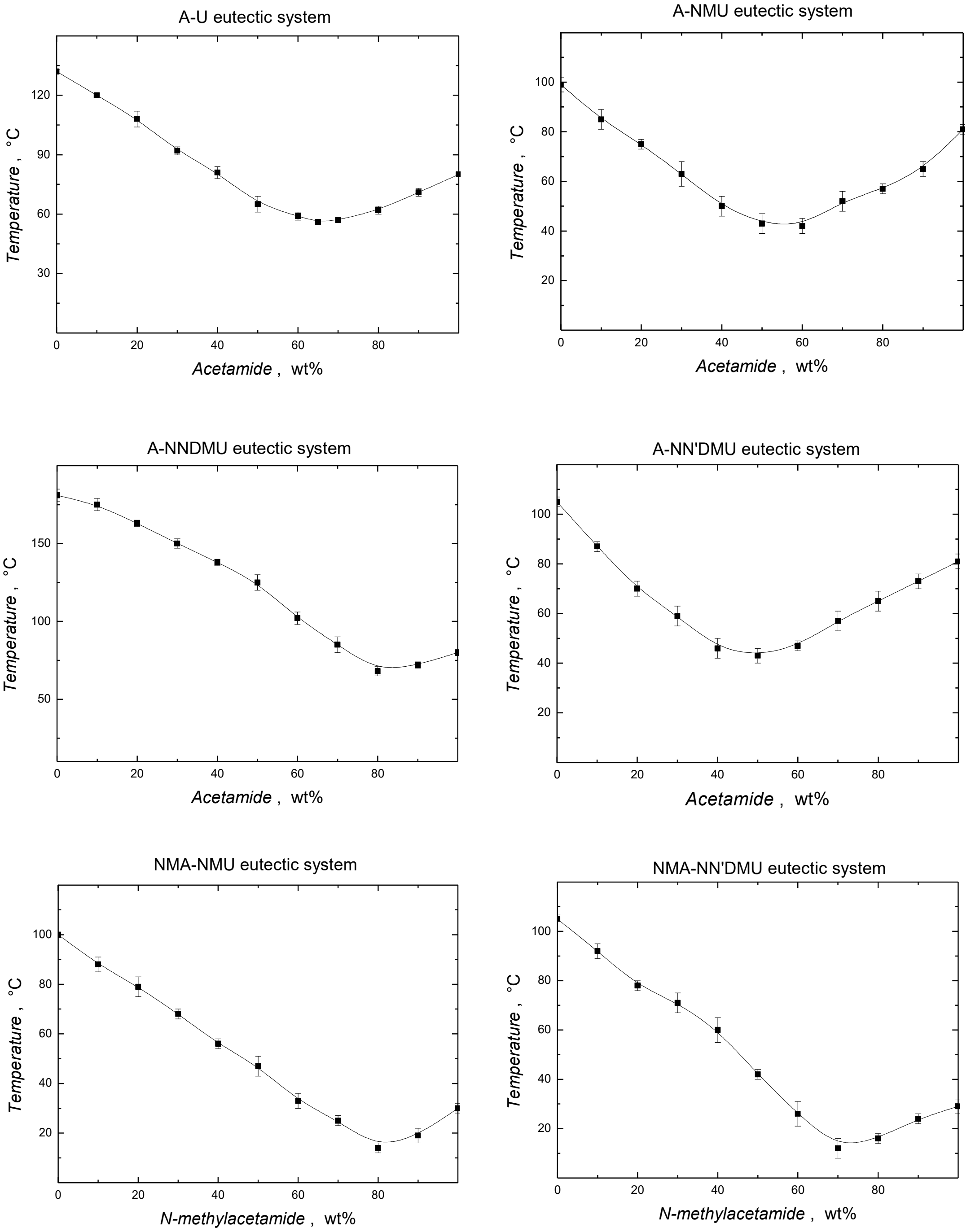

Figures S1. continued on the next page….

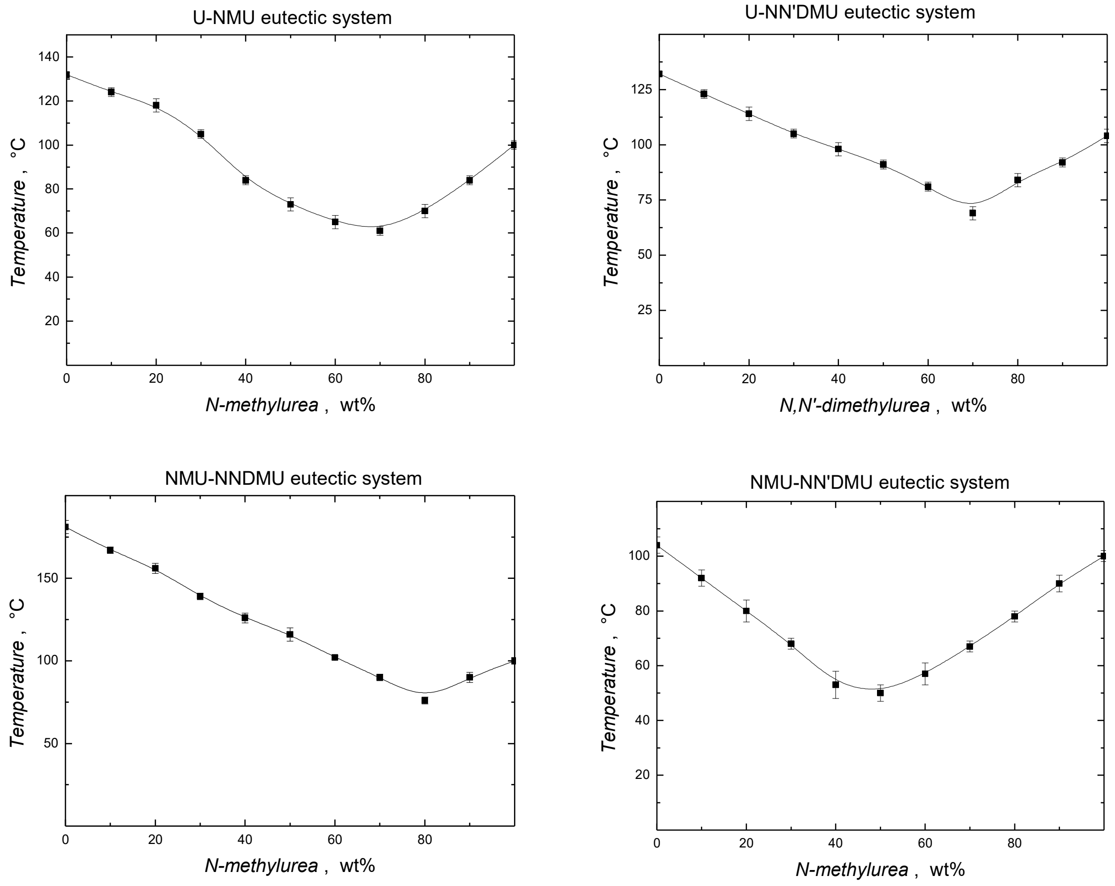

Figure S1 (continued from above). Phase diagrams of eutectic systems developed in this study.

(A)

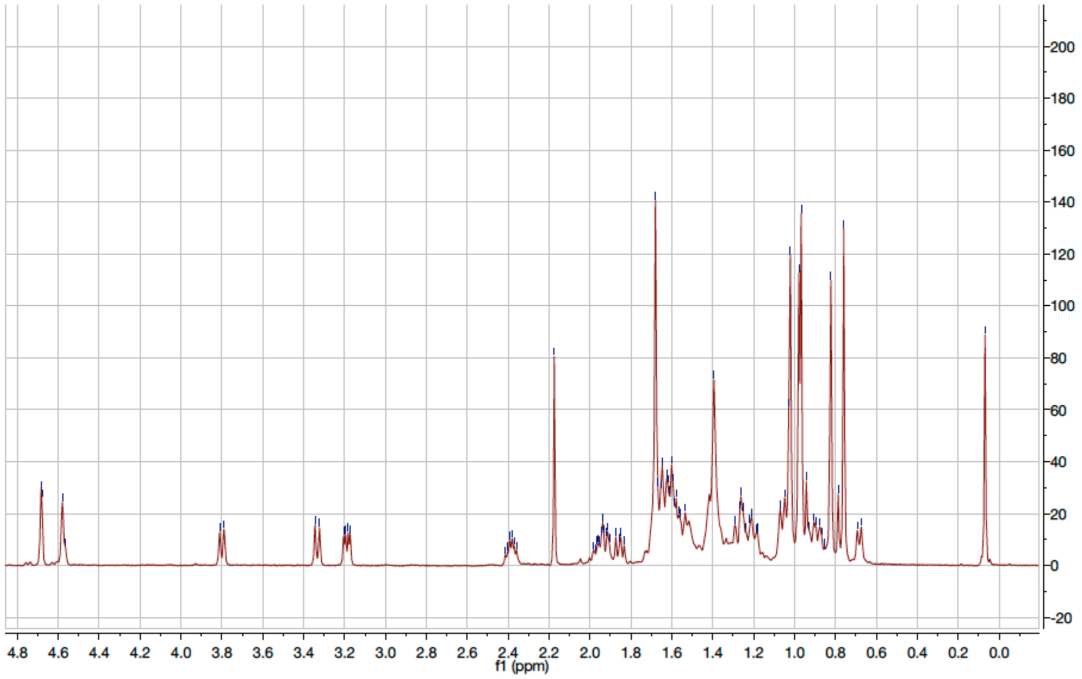

(B)

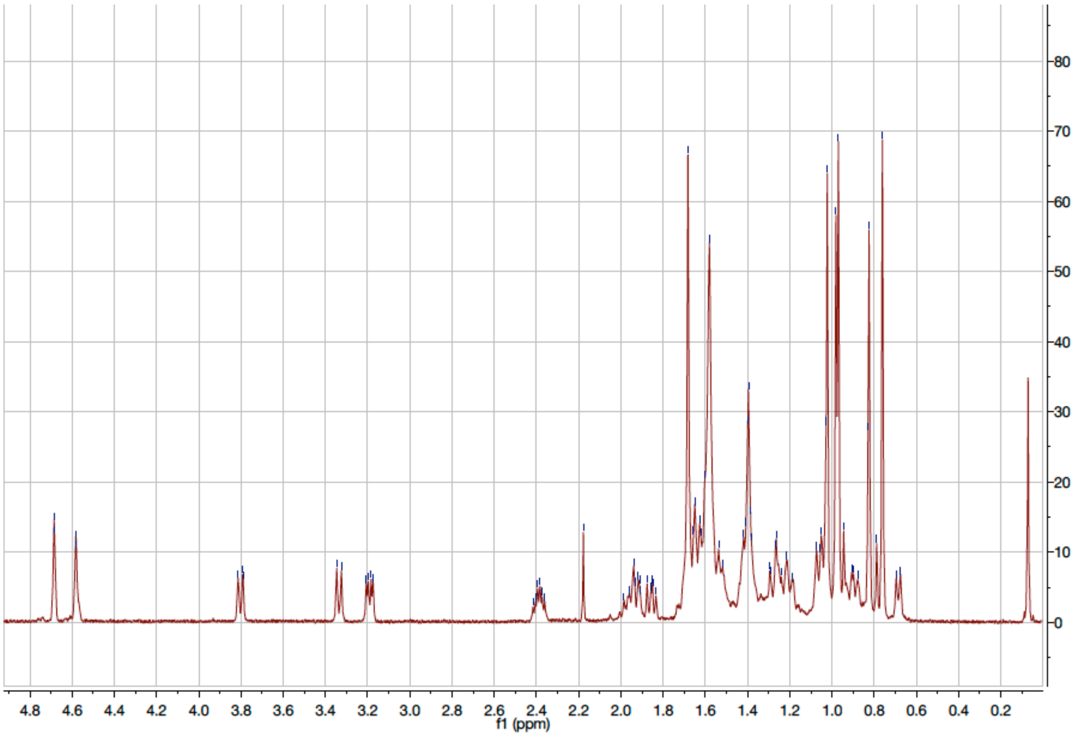

Figure S2. ^{1}H NMR spectra of betulin extracted from birch bark using (A) chloroform and (B) NMA-NMU eutectic system.

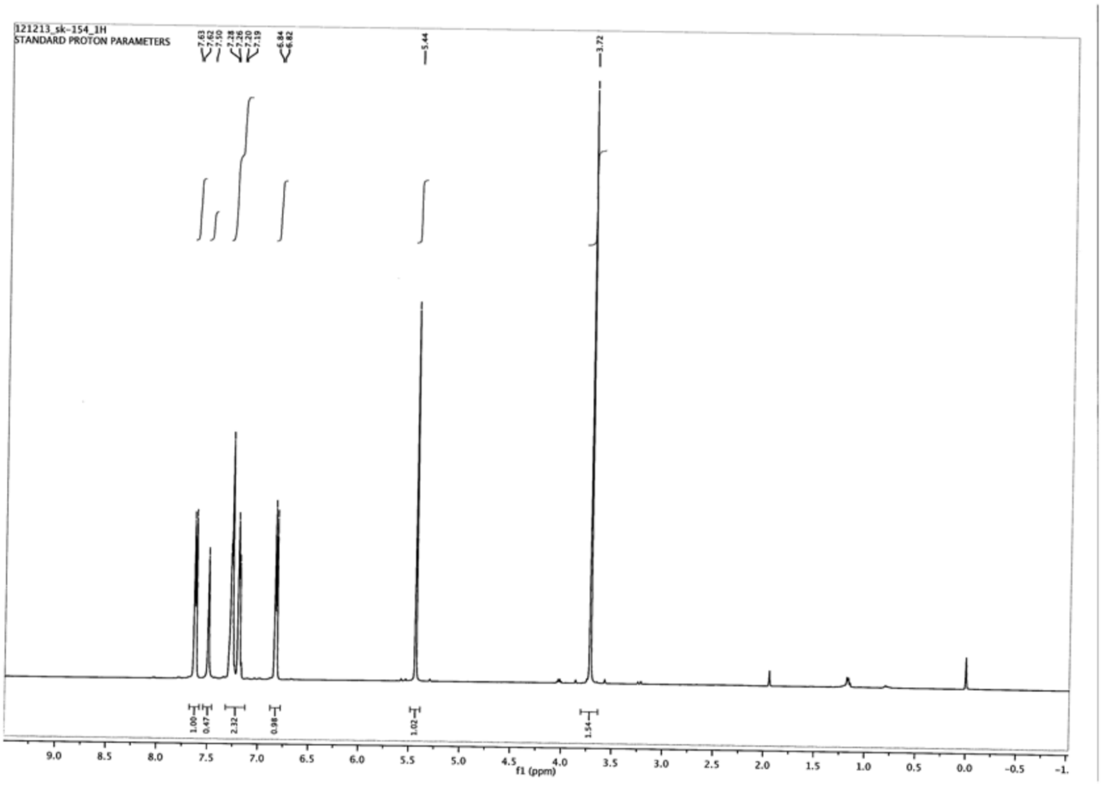

(A)

(B)

Figure S3. ^{1}H and ^{13}C NMR spectra of compound **5**

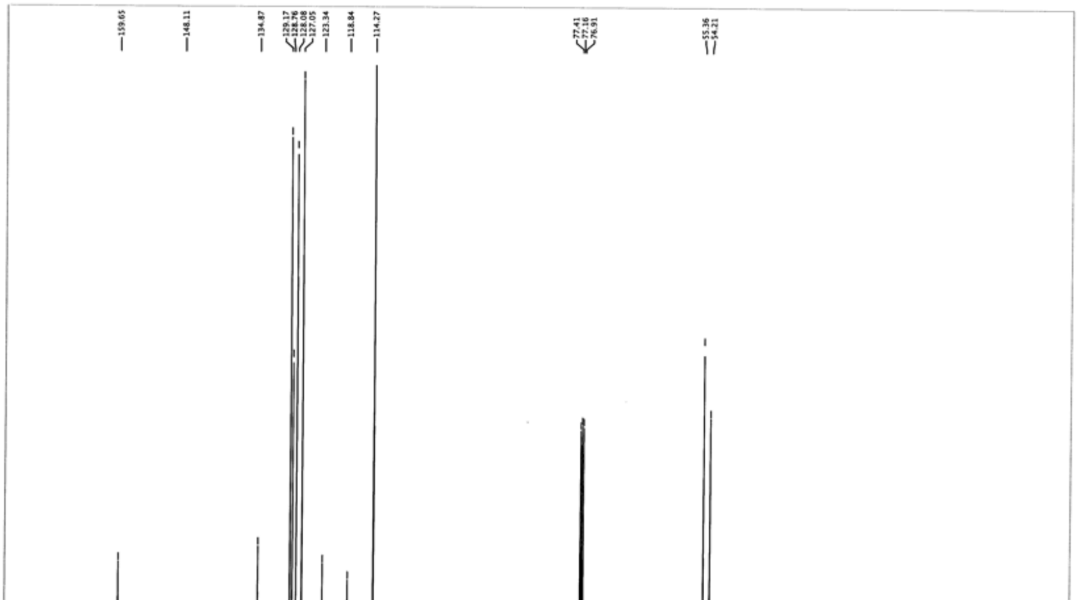

Table S1. Averaged hydrogen bond occupancies for A-U system at 343K, perspective acetamide (A)

		A					U			
		H	H1	H2	H3	H4	H1	H2	H3	H4
A	N	n.d.	n.d.	n.d.	0.184	0.390	0.214	0.132	0.124	0.220
	O	0.006	0.007	0.007	23.529	16.186	8.870	11.089	11.048	8.797
U	N1	n.d.	n.d.	n.d.	0.110	0.193	0.204	0.143	0.130	0.211
	N2	n.d.	n.d.	n.d.	0.108	0.196	0.212	0.123	0.137	0.221
	O1	0.002	0.004	0.002	14.541	10.182	10.164	14.450	14.555	10.389

All detected interactions were summarized and then averaged against the number of acetamide molecules in the system, with the exception of urea-urea interactions where the occupancy was averaged against the number of urea molecules in the system. n.d. = not detected

Table S2. Averaged hydrogen bond occupancies for A-U system at 343K, perspective urea (U)

		A					U			
		H	H1	H2	H3	H4	H1	H2	H3	H4
A	N						0.398	0.245	0.231	0.409
	O						16.474	20.593	20.518	16.337
U	N1	n.d.	n.d.	n.d.	0.203	0.359				
	N2	n.d.	n.d.	n.d.	0.200	0.364				
	O1	0.004	0.008	0.005	27.005	18.910				

All detected interactions were summarized and then averaged against the number of urea molecules in the system, with the exception of greyed out areas, where acetamide-acetamide interactions were averaged against the number of acetamide molecules and the urea-urea interactions would produce the same results as in Table 1 and are therefore not presented here. n.d. = not detected
